# An Unusual Presentation of Leptospirosis: A Case of Septic Shock and Proteinuria

**DOI:** 10.7759/cureus.64982

**Published:** 2024-07-20

**Authors:** Nismat Javed, Paul Kelly, Misbahuddin Khaja

**Affiliations:** 1 Internal Medicine, BronxCare Health System, Icahn School of Medicine at Mount Sinai, Bronx, USA; 2 Infectious Disease, BronxCare Health System, Icahn School of Medicine at Mount Sinai, Bronx, USA; 3 Internal Medicine/Pulmonary Critical Care, BronxCare Health System, Icahn School of Medicine at Mount Sinai, Bronx, USA

**Keywords:** proteinuria, complications, diagnosis, management, leptospirosis

## Abstract

Leptospirosis is a global health concern, particularly in tropical regions, with clinical symptoms varying from mild fever to severe organ dysfunction. We present a case of a 57-year-old male with septic shock and acute kidney injury due to acute leptospirosis. The patient's rapid progression to shock within a day of generalized symptoms was unusual. The patient's infection ultimately resolved with ceftriaxone and he was discharged after 14 days of therapy. The pathogenesis of severe leptospirosis is believed to be due to vasculitis, with organ damage caused by the leptospira bacteria and immune-mediated mechanisms. Diagnostic investigations include blood cultures and polymerase chain reactions, which are beneficial for early diagnosis. The management of patients depends on the severity of symptoms and other health conditions, as well as antibiotics and hydration. However, leptospirosis can lead to a wide range of complications, including neurological, ocular, hematological, and gastrointestinal involvement, necessitating vigilant monitoring and management.

## Introduction

Leptospirosis is a cause of global concern. The incidence of the disease varies from 0.10 to 975.00 cases annually per 100,000 population [1]. Disease rate was also significantly elevated in rural populations and tropical regions compared to urban settings [1]. It is predominantly found in tropical and sub-tropical regions [2]. Each year, it is projected that over 1 million cases occur worldwide, resulting in nearly 60,000 fatalities [2]. In the United States, the annual reported cases of leptospirosis range from around 100 to 150 [2]. Most of these cases are reported in Puerto Rico, with Hawaii following closely [2]. Clinical symptoms of leptospirosis in humans can vary widely, from a mild fever that resolves on its own to a severe, life-threatening condition involving multiple organ dysfunction [3]. While most patients present with a simple fever, around 10% develop severe disease [3]. The classic presentation of Weil's syndrome includes conjunctival suffusion, jaundice, and acute kidney injury. Recently, pulmonary hemorrhage has been identified as a significant cause of death [3]. Antimicrobial agents form the cornerstone of management. However, source control also has a role in active and passive surveillance [3]. Here, we present the case of a 57-year-old male who presented with septic shock and acute kidney injury secondary to acute leptospirosis. The case was unusual given the patient's quick progression to shock and the development of proteinuria.

## Case presentation

A 57-year-old male presented to the emergency department with an 8-hour history of generalized weakness. The complaints worsened one day prior to presentation, after consuming sandwiches. The complaints were associated with epigastric pain, two episodes of diarrhea, and one episode of non-bilious and non-blood-stained vomiting. The complaints did not resolve after taking non-steroidal anti-inflammatory (NSAID) agents or after using antibiotics, specifically azithromycin. The patient also mentioned subjective fever that did not resolve with antipyretics. He denied any headache, chest pain, abdominal pain, nausea, vomiting, or burning micturition.

Vitals on presentation were significant for fever (101.3 degrees F), tachycardia (141 beats per minute), and hypotension (89/68 mmHg). Physical examination revealed mild epigastric tenderness. Labs on admission were significant (Table 1) for elevated creatinine (3.4 mg/dl; reference range: 0.5-1.5 mg/dl) while urinalysis revealed proteinuria as well as leukocyte esterase, mild transaminitis, and elevated lactic acid (4.5 mmoles/L; reference range: 0.5-1.6 mmoles/L).

**Table 1:** Initial laboratory investigations

Investigation	Result	Normal Range
Hemoglobin (g/dl)	14.0	12.0-16.0
WBC (/uL)	16100	4800-10800
Platelets (/uL)	119000	150000-400000
Sodium (mEq/L)	134	135-145
Potassium (mEq/L)	4.1	3.5-5.0
Calcium (mEq/L)	8.8	8.5-10.5
Chloride (mEq/L)	94	98-108
Bicarbonate (mEq/L)	19	24-30
Blood Urea Nitrogen (mg/dl)	47.0	6-20
Creatinine (mg/dl)	3.4	0.5-1.5
Total Bilirubin (mg/dl)	1.6	0.2-1.2
Direct Bilirubin (mg/dl)	1.0	0.5-1.5
Alanine Aminotransferase (unit/L)	52	5-40
Aspartate Transaminase (unit/L)	92	9-48
Alkaline Phosphatase (unit/L)	144	56-119

**Figure 1:**
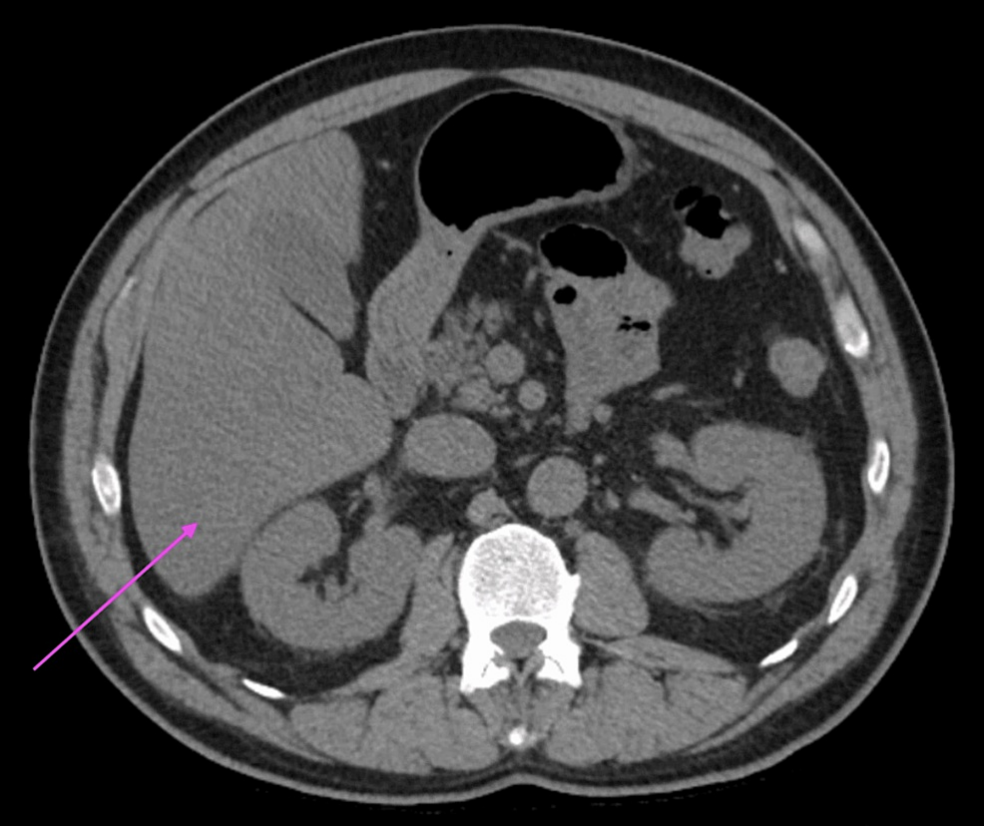
CT abdomen revealed fatty infiltration of the liver (marked by the pink arrow), a small hiatal hernia, and a 1.6-cm right adrenal mass

CT abdomen revealed fatty infiltration of the liver, a small hiatal hernia, and a 1.6-cm right adrenal mass (Figure 1).

He was given 3 liters of normal saline in the emergency department as well as empirical antibiotics (intravenous vancomycin and piperacillin-tazobactam). Urine and blood cultures came back negative for any pathogen. However, blood pressure failed to improve. ICU was consulted for septic shock, and he was transferred.

Nephrology was consulted and differential diagnoses of prerenal acute kidney injury and ischemic acute tubular necrosis were considered. Urine electrolytes revealed fractional excretion of urea at 37.3% and fractional excretion of sodium at 5.2%. Ultrasound of the abdomen did not show any renal disease. However, in view of worsening kidney function on day 2 of hospitalization (creatinine: 5.1 mg/dl; reference range: 0.5-1.5 mg/dl), vancomycin was held. However, he started developing worsening white blood cell (WBC) counts (increased to 19500/uL) and thrombocytopenia (decreased to 100000/uL) on day 2 of hospitalization. Infectious diseases were also consulted and piperacillin-tazobactam was continued. On day 6 of hospitalization, leptospira antigen in urine came back positive, and intravenous piperacillin-tazobactam was switched to ceftriaxone as per infectious disease recommendations. As a result, his leukocytosis (decreased to 10900/uL) and creatinine (decreased to 2.1 mg/dl) started to improve on day 8 of hospitalization. He was discharged after 14 days of hospitalization and completion of antibiotics. He is also scheduled to follow up with the outpatient clinic for infectious diseases and nephrology.

## Discussion

Acute leptospirosis has been previously described in the literature. Septic shock, although associated with the disease, is an uncommon presenting feature of the disease. The average age of the patients with this clinical presentation varies from 40 to 70 years of age, as was observed in our case [4-7]. The incubation period for leptospirosis can range from 2 to 20 days, with most cases occurring within 7 to 12 days [3]. Some patients may experience a biphasic illness, with the disease course traditionally divided into an acute 'leptospiraemic phase' and a subsequent 'immune phase' [3]. The initial phase often presents as a non-specific acute fever, with symptoms such as fever, chills, muscle pain, and headache [3]. This is followed by an immune phase, where antibodies appear in the blood and the organisms are excreted in the urine [3]. Serious complications can occur during this stage, depending on the extent of organ involvement and the organism's virulence [3]. As a consequence, septic shock might develop at this stage. In our case, shock development was relatively rapid, within a day of generalized symptoms.

The exact pathogenesis of severe leptospirosis remains unclear, but it is believed to be due to a type of vasculitis [3]. Similar to other bacterial infections, tissue and organ damage and alterations in tissue microcirculation and endothelial function are caused by both direct harm from the leptospira bacteria and immune-mediated mechanisms [3]. While jaundice is a prominent symptom, death often occurs due to complications from acute kidney injury, heart involvement, or pulmonary hemorrhage. Severe manifestations that can lead to high mortality rates include severe pulmonary involvement, hemorrhagic symptoms, and myocarditis [3].

The clinical spectrum usually varies from a limited febrile illness to worsening symptoms, as observed in our case. Common symptoms include fever, headache, and myalgias [4]. Severe features can consist of icterus or hemoptysis on presentation [5]. Usually, most of the cases present with hemodynamic instability on presentation in such a cohort of patients [4-7]. Lab investigations could reveal leukocytosis, lactic acidosis, worsening transaminitis, and worsening kidney function [4-7]. While our case had all the above findings, proteinuria observed in urinalysis was unusual and suggested an underlying possibility of chronic kidney disease.

Diagnostic investigations include blood cultures and polymerase chain reaction. Although blood cultures have an essential role in determining antibiotic sensitivity, they are usually positive during the first week of illness [3,8]. Polymerase chain reaction is beneficial for early diagnosis during the first week of acute illness [8]. It offers high sensitivity and specificity and is helpful for genomic classification. Antibodies are typically detectable by the sixth to tenth day of sickness and peak within three to four weeks [9]. The microscopic agglutination test is the serological reference standard [3]. However, a great degree of experience is needed to minimize any variations in the interpretation of the test [3]. It is time-consuming and hazardous, requiring live cultures to provide the antigen [3]. This test is only available in reference laboratories. The immunoglobulin M (IgM) enzyme-linked immunosorbent assay (ELISA) is readily available [10]. Its sensitivity and specificity depend on regional patterns of seropositivity [10].

Patients with suspected or confirmed leptospirosis who exhibit mild clinical symptoms and have no additional health conditions may be managed as outpatients, with regular check-ups to identify potential complications [11]. However, patients displaying organ involvement or those with other health conditions should be admitted for in-patient care. While initiating antibiotics early can improve prognosis, mortality benefit is still being investigated [12]. For mild infections, oral doxycycline is suggested as a first-line treatment. Intravenous antibiotics, such as penicillin or ceftriaxone, are warranted in cases of septic shock [13]. Adequate hydration is critical to managing patients with concomitant renal injury [3]. High-dose corticosteroids have a controversial role in severe leptospirosis and are not supported by high-quality evidence for treating leptospirosis [14]. Plasmapheresis has also been used in severe leptospirosis cases [15].

A wide range of associated complications can occur, including acute disseminated encephalomyelitis, hydrocephalus, increased intracranial pressure, coma induced by encephalitis, intracranial vascular events, intracranial bleeding, thrombosis, cerebellar syndrome, transverse myelitis, Guillain-Barré syndrome, and various types of neuritis [3]. Ocular manifestations, such as uveitis, optic neuritis, and retinal phlebitis, have been reported [3]. There can also be hematological involvement, resulting in pancytopenia, hemolytic anemia, hemolytic uremic syndrome, and thrombotic thrombocytopenic purpura [3]. Gastrointestinal involvement can lead to conditions like pancreatitis and cholecystitis [3]. Acute kidney injury and proteinuria have been recognized as possible complications of the infection in some case reports, however, literature regarding these presentations is limited [16,17].

Early recognition and management of the disease is crucial. Additionally, patients with chronic kidney disease should be considered at high risk for the progression of renal dysfunction and the development of proteinuria. There is a need for further studies to better understand the cohort of patients who have chronic kidney disease, which worsens with leptospirosis.

## Conclusions

Leptospirosis, commonly presenting as a febrile illness, can rapidly progress to serious conditions such as septic shock. Diagnostic approaches like blood cultures and PCR are crucial, and early antibiotic treatment can improve prognosis. The disease can lead to diverse complications, necessitating comprehensive patient management and monitoring.
